# Hexokinase 2 Is a Pivot for Lovastatin-induced Glycolysis-to-Autophagy Reprogramming in Triple-Negative Breast Cancer Cells

**DOI:** 10.7150/jca.71592

**Published:** 2022-09-21

**Authors:** Lian Xue, Mi Wu, Ying Li, Sisi Chen, Muyao Wu, Jianyu Zhu, Siyu Ding, Qiuting Zhang, Chanjuan Zheng, Guangchun He, Shujun Fu, Guifei Li, Xiyun Deng

**Affiliations:** 1 Key Laboratory of Translational Cancer Stem Cell Research, Department of Pathophysiology, Hunan Normal University School of Medicine, Changsha, Hunan 410013, China.; 2 Department of Biochemistry and Molecular Biology, Jishou University, Jishou, Hunan, China.; 3 Department of Nursing, Hunan Normal University School of Medicine, Changsha, Hunan 410013, China.

**Keywords:** Lovastatin, Triple-negative breast cancer, Metabolomics, Glycolysis, Autophagy

## Abstract

Triple-negative breast cancer (TNBC) is the most aggressive subtype of breast cancer with limited therapeutic options available. We have recently demonstrated that lovastatin, a 3-hydroxy-3-methylglutaryl-coenzyme A (HMG-CoA) reductase inhibitor, suppresses TNBC cell proliferation and stemness properties* in vitro* and* in vivo*. However, the mechanisms through which lovastatin inhibits TNBC cells are not fully understood. Here, we used ^1^H NMR-based metabolomic profiling to investigate lovastatin-induced metabolic changes in TNBC cell line MDA-MB-231. Among the 46 metabolites identified, lactate demonstrated the highest variable importance in projection (VIP) score. Glycolysis stress test revealed that lovastatin significantly decreased the extracellular acidification rate (ECAR) in MDA-MB-231 cells. Furthermore, lovastatin treatment down-regulated the levels of glycolysis-related proteins including GLUT1, PFK1, and PKM2 in MDA-MB-231 but not non-TNBC MDA-MB-453 cells. In addition, lovastatin induced autophagy as evidenced by increased LC3 puncta formation and LC3-II/I ratio, increased AMPK phosphorylation, and decreased Akt phosphorylation. We also revealed the interaction between the glycolytic enzyme hexokinase 2 (HK2) and the mitochondrial membrane protein voltage-dependent anion channel 1 (VDAC1), an important regulator of autophagy. Further bioinformatics analysis revealed that VDAC1 was expressed at a higher level in breast cancer than normal tissues and higher level of VDAC1 predicted poorer survival outcomes in breast cancer patients. The present study suggests that lovastatin might exert anti-tumor activity by reprogramming glycolysis toward autophagy in TNBC cells through HK2-VDAC1 interaction.

## Introduction

In spite of the progress in screening and early diagnosis, breast cancer remains the most common female malignancy globally 1. Characterized by the lack of estrogen receptor (ER), progesterone receptor (PR), and human epidermal growth factor receptor 2 (HER2), TNBC accounts for 10-20% of all breast cancer cases and has a median overall survival time of less than 18 months in the metastatic setting 2. Although targeted therapeutic strategies have been extensively explored in TNBC, the efficacy is still limited for various reasons including the occurrence of treatment-related adverse events, etc 3. Therefore, it is a very urgent task to explore novel therapeutic drugs for TNBC.

Even in the presence of abundant oxygen, a majority of tumor cells produce substantial amounts of energy through enhanced glycolytic metabolism rather than oxidative phosphorylation. This phenomenon, known as the Warburg effect 4, has been well recognized in a variety of solid cancer types. The enzymes and transporters involved in the aerobic glycolytic pathway, in association with the intracellular signaling pathways, play critical roles in metabolic reprogramming of cancer cells. Over the last decade, there has been immense interest in targeting key glycolysis-related enzymes such as hexokinases (HKs), phosphofructokinases (PFKs), pyruvate kinases (PKs), and glucose transporters (GLUTs), as a possible avenue for cancer therapy. Since TNBC has well-known dysregulation in glucose metabolism 5, agents targeting the molecules involved in the hyperactivity of glycolysis may provide a potential therapeutic option for this type of hard-to-treat cancer.

Statins, 3-hydroxy-3-methylglutaryl-coenzyme A (HMG-CoA) reductase inhibitors, are widely used as lipid-lowering drugs due to their advantages of low adverse events and pleiotropic effects other than HMG-CoA reductase inhibition 6. Lovastatin is the first US FDA-approved naturally occurring lipophilic statin, whose anti-tumor effect has gained enormous attention. In clinical trials, the risk of developing breast cancer has been shown to be reduced after long-term use of lovastatin 7. Mechanistically, lovastatin can inhibit proliferation, migration, and invasion and induce apoptosis* in vitro* and* in vivo* in a variety of cancer types including breast cancer 7, 8. Recently, we have demonstrated that lovastatin could inhibit the stemness properties, reverse the HER2-negative phenotype, and inhibit metastasis of TNBC cancer stem cells in preclinical models 9-11. However, how lovastatin exerts its inhibitory effects on TNBC remains largely elusive.

The connection between glycolysis and autophagy has been an area of intense investigation in recent years. It has been demonstrated that glycolysis regulates autophagy through multiple mechanisms, which plays a critical role in the acquisition of malignant phenotypes of cancer cells 12. A group of mitochondrial pore-forming proteins, known as voltage-dependent anion channels (VDACs), are critical for the maintenance of mitochondrial integrity. Located at the interface between the cytosol and the mitochondria, VDACs are the docking sites for several proteins important for autophagy regulation. VDAC1, the most abundant and best characterized VDAC isoform, has become a new pharmacologically targetable molecule for cancer therapy 13. Autophagy induction by statins has been documented in several types of cancer cells including melanoma, prostate, and breast cancer 14-16. Recently, we have demonstrated that lovastatin promotes the formation of autophagosomes in TNBC cancer stem cells (unpublished observation). However, it is not known whether lovastatin induces autophagy through the regulation of metabolic reprogramming. In this study, we found that lovastatin exerts anti-tumor activity by reprogramming glycolysis toward autophagy in TNBC cells. Our findings provide rationale for designing a novel therapeutic strategy based on the connection between glycolysis and autophagy in TNBC.

## Materials and Methods

### Antibodies

Antibodies against HK2 and VDAC1 were purchased from Santa Cruz Biotechnology (Santa Cruz, CA, USA) and Proteintech (Chicago, IL, USA), respectively. Antibodies against LC3, mTOR, mTOR^Ser2448^, AMPK, AMPK^Thr172^ and, Akt^Ser473^ were purchased from Cell Signaling Technology (Danvers, MA, USA). Antibodies against Tubulin and GAPDH were purchased from Bioworld Technology (Philadelphia, PA, USA) and Zhongshan Golden Bridge (Beijing, China), respectively. Anti-rabbit IgG (H+L) affinity purified secondary antibody was obtained from Vector Laboratories (Burlingame, CA, USA).

### Cell culture and reagents

MDA-MB-231, MDA-MB-468, and MDA-MB-453 human breast cancer cells were purchased from the Cell Resource Center of Shanghai Institutes for Biological Sciences, maintained in Dulbecco's high glucose (25 mM glucose) modified Eagle's medium (DMEM) supplemented with 10% fetal bovine serum (FBS) (Gibco, Carlsbad, CA, USA), 100 U/mL penicillin, and 100 μg/mL streptomycin. The cells were cultured at 37 °C in a humidified incubator with 5% CO_2_. Lovastatin was purchased from Sigma (M2147, St Louis, MO), dissolved in DMSO as a stock solution of 30 mM, which was aliquoted and stored frozen at -20 °C.

### Cell treatment

MDA-MB-231, MDA-MB-468, and MDA-MB-453 human breast cancer cells were seeded in culture dishes or plates (about 1.5 × 10^4^ cells/cm^2^) and allowed to grow overnight before treatment. On the next day, lovastatin was added to the cells at various concentrations and the cells were cultured under normoxia (21% O_2_) or hypoxia (1% O_2_) for the desired period of time as previously described 17.

### ^1^H NMR-based metabolomic profiling

^1^H nuclear magnetic resonance (NMR) analysis based on metabolite identification was conducted as previously described 18. Following the normalization processing, independent-sample* t* test was further used to investigate alterations in endogenous metabolites using SPSS (version 13) software. The peaks with adjusted *P* values < 0.05 were deemed statistically significant. Principal component analysis (PCA) and partial-least-squares discriminant analysis (PLS-DA) were performed using the R statistical package. Data was visualized by employing the ggplot2. The PCA scores plot was used to reveal the metabolomic profiles of lovastatin-treated cells. The influence of variables was predicted by their variable importance in projection (VIP) scores. A VIP score above 1.00 was considered statistically significant.

### Measurement of glucose uptake and lactate production

The cells were seeded in six-well plates (2 × 10^5^, 8 × 10^5^, and 4 × 10^5^ cells per well for MDA-MB-231, MDA-MB-468, and MDA-MB-453, respectively) in triplicate wells and incubated overnight. On the next day, various concentrations of lovastatin (0, 1, and 3 μM) were added to the medium and the culture was continued for up to 48 h. The medium was collected, and glucose and lactate levels were then measured using the Automatic Biochemical Analyzer (AU680, Beckman, USA) at the Clinical Biochemical Laboratory of Xiangya Hospital, Central South University. The protein concentration of the cells in each well were measured and used for normalization.

### Glycolysis stress test

The cells were seeded on XF24 multi-well plates (2 × 10^4^, 8 × 10^4^, and 5 × 10^4^ cells per well for MDA-MB-231, MDA-MB-468, and MDA-MB-453, respectively) in triplicate and were pre-incubated overnight at 37°C with 5% CO_2_, followed by incubation with various concentrations of lovastatin (0, 1, and 3 μM) for 48 h. The glycolysis stress test was performed in the Seahorse XF24 Extracellular Flux Analyzer (Seahorse Bioscience, USA) according to the manufacturer's protocol. The relative levels of glycolysis, glycolytic capacity and glycolytic reserve were calculated based on extracellular acidification rate (ECAR) data. The data were normalized by the protein concentration in each well.

### Western blotting

After lovastatin (0, 1 and 3 μM) treatment, MDA-MB-231 and MDA-MB-453 cells were harvested and lysed in cell lysis buffer with PMSF and phosphatase inhibitors added. The protein concentration was determined and equal amounts of protein from each lysate were run on the SDS-PAGE gel. After electrophoretic separation, the proteins were transferred to a PVDF membrane, followed by primary and secondary antibody incubation and ECL development according to our standard procedure 11. The protein bands were quantified using the ImageJ software.

### Immunofluorescence-confocal microscopy

The cells were grown on coverglasses and treated with lovastatin or vehicle for 48 h as described 11. Then the cells were fixed with 4% paraformaldehyde and subjected to indirect immunofluorescence. The cells were imaged using a Leica TCS SP8 Confocal Microscope (Wetzlar, Germany). Bidirectional scanning and 2× line averaging was used on a 2,048 × 2,048 resolution with zoom adjusted according to the field of interest.

### Co-immunoprecipitation (co-IP) assay

After treatment with lovastatin, the cells were lysed in 1× cell lysis buffer (Cell Signaling Technology, Danvers, MA, USA) with 1 mM phenylmethanesulfonyl fluoride (Sigma, St Louis, MO, USA) and phosphatase inhibitors (Thermo Fisher Scientific, Waltham, MA, USA). Co-immunoprecipitation between HK2 and VDAC1 was carried out using the Dynabeads Protein G Immunoprecipitation Kit (Thermo Fisher Scientific, Waltham, MA, USA) following the manufacturer's protocol.

### Hexokinase activity assay

The cells were seeded into a 6-well plate and grown for 24 h, and then incubated with different concentrations of lovastatin for 24 h. The cells were counted and cell pellets were obtained by centrifugation and then lysed with 1× lysis buffer (Cell Signaling Technology, Danvers, MA, USA) containing 1 mM PMSF (Sigma, St Louis, MO, USA) and phosphatase inhibitors (Thermo Fisher Scientific, Waltham, MA, USA). The protein concentration in cell lysate was determined by BCA assay, and the HK activity in cell lysate was analyzed with Micro HK Assay Kit (Solarbio, Beijing, China) following the instructions of the manufacturer.

### Transmission electron microscopy

Autophagosomes and autolysosomes were observed by routine transmission electron microscopy (TEM). Briefly, the cells were treated with vehicle or lovastatin (1 μM) for 24 h, fixed, post-fixed, and subjected to embedding, sectioning, and TEM observation as described in our recent publication 11.

### Bioinformatics analysis

The breast cancer gene expression and patient annotation datasets of The Cancer Genome Atlas (TCGA) were retrieved from the Genomic Data Commons (GDC) (https://portal.gdc.cancer.gov/) of the National Cancer Institute. Gene set enrichment analysis (GSEA) (https://www.gsea-msigdb.org/gsea/msigdb/index.jsp) was performed using the GSEA 4.1.0 program 19. The differentially expressed genes were obtained by dividing breast cancer patients into high and low expression groups according to the median of HK2 gene expression level. The correlation of HK2 expression with the autophagy-related markers in breast cancer was analyzed using the online database Gene Expression Profiling Interactive Analysis 2 (GEPIA2) (http://gepia2.cancer-pku.cn/#index) 20. Protein-protein interaction (PPI) network generated using the STRING database (https://string-db.org/) 21 was applied to analyze the functional groups of autophagy-related proteins in breast cancer. UALCAN (http://ualcan.path.uab.edu/) 22 was applied to analyze the transcriptional levels of HK2 and VDAC1 in primary breast cancer tissues from TCGA database. The prognostic value of HK2 and VDAC1 in TNBC patients were analyzed using Kaplan-Meier plotter (http://kmplot.com/analysis/).

### Statistical analysis

All the quantitative data were presented as mean ± SEM. Statistical analyses (ANOVA, unpaired Student's t test) were carried out using SigmaPlot (version 12.5). *P* < 0.05 was considered as statistically significant.

## Results

### Lovastatin alters the metabolomic profile in TNBC cells

**Figure 1:**
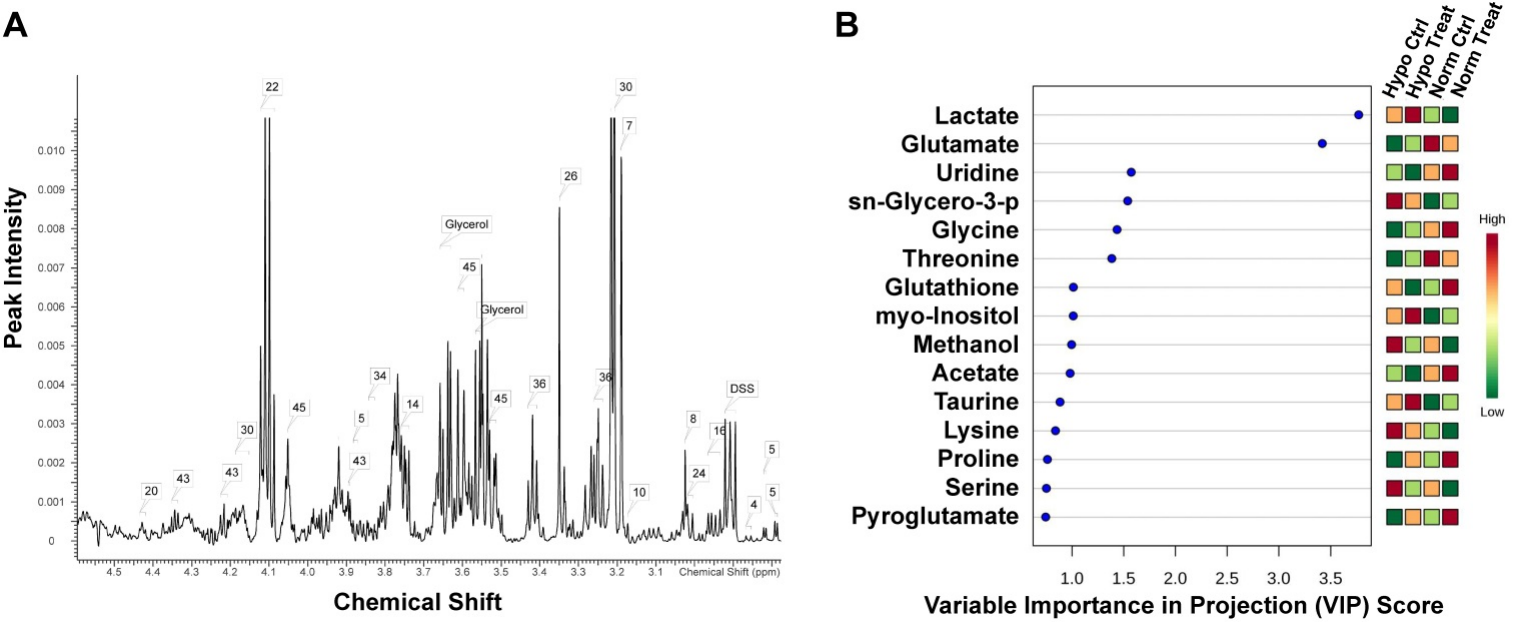
** Lovastatin alters the metabolomic profile in TNBC MDA-MB-231 cells.** (**A**) ^1^H NMR-based metabolomics detected in 15-cm culture dishes for 72 h treated with lovastatin in MDA-MB-231 cells. The attribution range was 3.0ppm_5.0ppm. The assigned metabolites were identified with a serial number. (**B**) The VIP scores depicting the 15 top-ranked metabolites that contributed to the separation of MDA-MB-231 breast cancer cells treated with lovastatin or DMSO. A VIP score above 1.00 was considered statistically significant. The colored boxes on the right indicate the relative concentrations of the corresponding metabolite in each group under study. The scale bar depicts the relative metabolite levels (red, high abundance; green, low abundance). Hypo: hypoxia; Norm: normoxia; Ctrl: control

In order to gain insight into the effect of lovastatin on metabolic reprogramming in breast cancer cells, we performed global metabolomic profiling using MDA-MB-231 as a representative TNBC cell line by an NMR-based metabolomics approach. The typical 500 MHz ^1^H NMR spectra with peak assignments for the extracts from MDA-MB-231 cells were shown in **Figure 1A**. The PCA score plot for ^1^H NMR data of MDA-MB-231 cells showed a clear separation between normoxia and hypoxia groups, with the lovastatin-treated group and the control group overlapping with each other (**Figure S1**). To get a better separation, partial least-squares discriminant analysis (PLS-DA) was performed to explore the differences in metabolomics among the treatment and control groups (**Figure S2**). Among the metabolites identified, marked decrease in the levels of lactate, glutamate, glycine, threonine, methanol, lysine, and serine was observed in the lovastatin-treated MDA-MB-231 cells compared with the control cells, with lactate being on the top list of VIP scores (**Figure 1B**).

### Lovastatin suppresses glycolysis but contrarily increases HK2 protein level in TNBC cells

**Figure 2:**
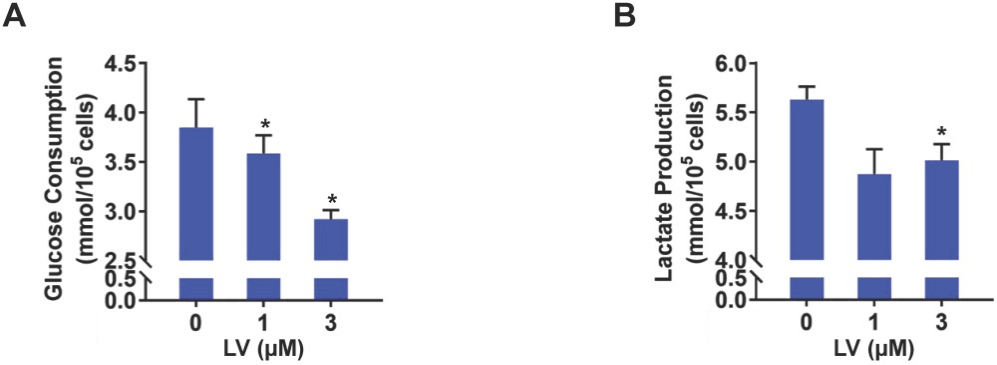
** Lovastatin decreases glucose consumption and lactate production in breast cancer cells.** (**A, B**) The levels of glucose consumption (**A**) and lactate production (**B**) examined in culture medium collected from control or lovastatin-treated (48 h) MDA-MB-231 cells. * *P* < 0.05, ** *P* < 0.01, compared with the control.

**Figure 3:**
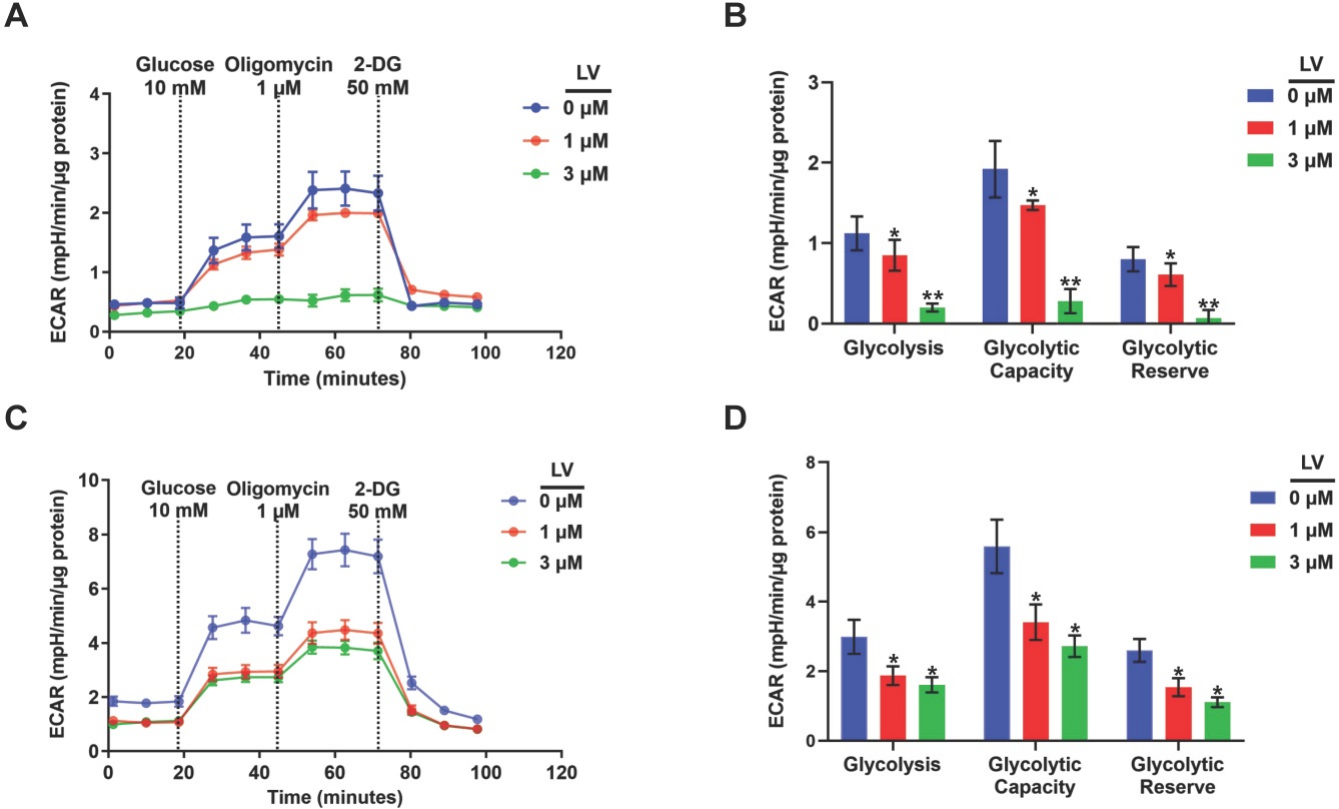
**Lovastatin decreases glucose metabolism in breast cancer cells.** (**A**) ECAR measured in MDA-MB-231 cells treated with vehicle, or lovastatin at 1 or 3 μM. (**B**) Quantifications of the ECAR results of MDA-MB-231 cells, showed glycolysis, glycolytic capacity, and glycolytic reserve, respectively. (**C**) ECAR measured MDA-MB-468 cells treated with vehicle, or lovastatin at 1 or 3 μM. (**D**) Quantifications of the ECAR results of MDA-MB-468 cells, showed glycolysis, glycolytic capacity, and glycolytic reserve, respectively. * *P* < 0.05, ** *P* < 0.01, compared with the control.

Based on the findings from metabolomic profiling showing a significant decrease in lactate level induced by lovastatin, we further examined the levels of glucose consumption and lactate production in control and lovastatin-treated cells. Consistent with the results of metabolomics analysis, less amount of glucose consumed from the medium (**Figure 2A**) and less amount of lactate secreted into the medium (**Figure 2B**) were observed in lovastatin-treated MDA-MB-231 cells compared with the control cells. However, lovastatin did not significantly influence glucose consumption and lactate production of HER2-positive MDA-MB-453 cells (**Figure S3**). Seahorse glycolysis stress test showed that lovastatin significantly decreased the ECAR associated with glycolysis in MDA-MB-231 (**Figure 3A, B**) and MDA-MB-468 (**Figure 3C, D**) cells.

**Figure 4:**
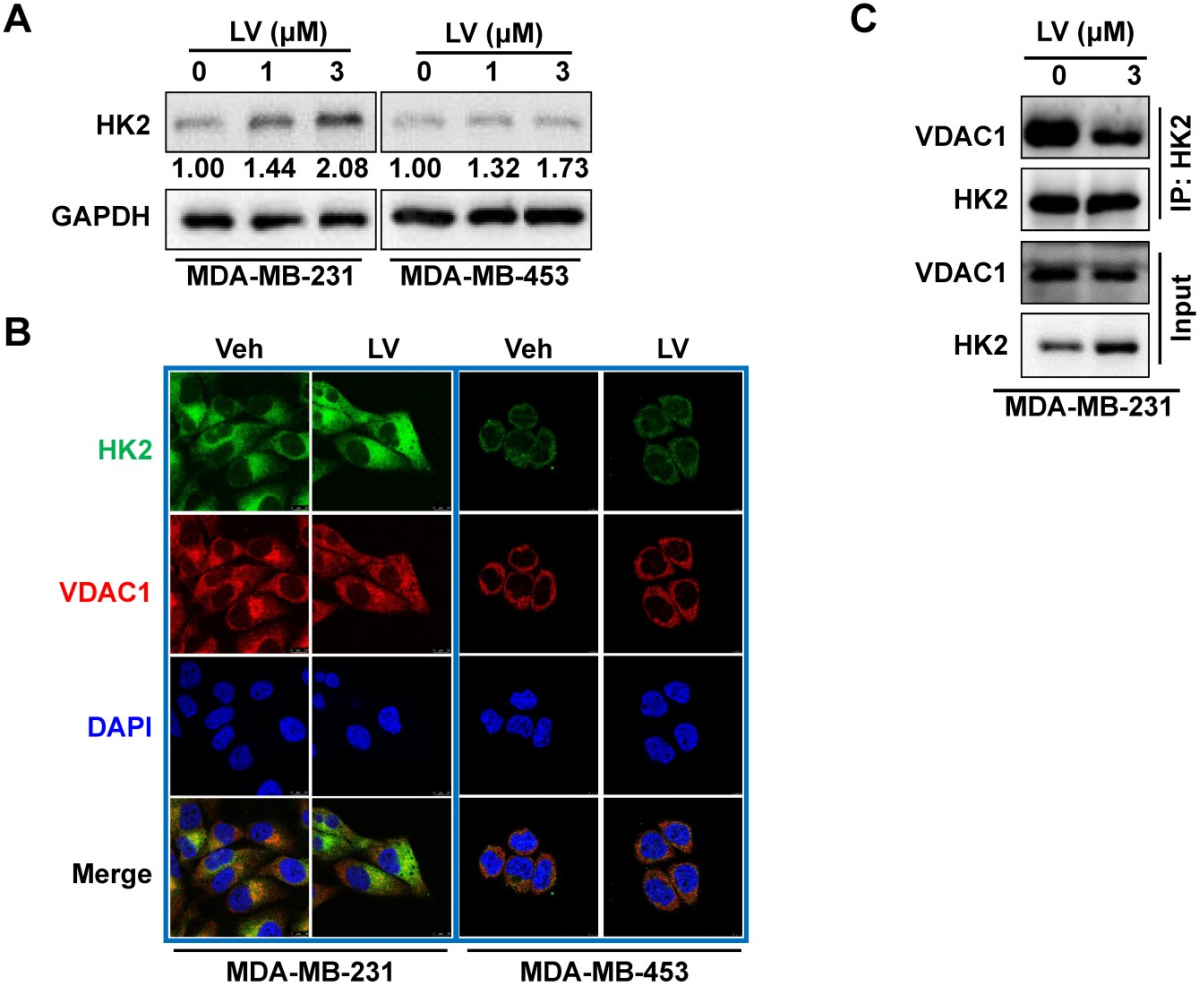
** Lovastatin decreases the interaction of VDAC1 and HK2 in TNBC cells.** (**A**) The protein level of HK2 analyzed by Western blotting in MDA-MB-231 cells treated with lovastatin or vehicle for 48 h. (**B**) Representative confocal images of immunofluorescence staining for HK2 (green) and VDAC1 (red) in MDA-MB-231 and MDA-MB-453 cells after treatment with lovastatin (3 μM, 48 h) or vehicle. Blue, DAPI staining of the nucleus. Original magnification: 630×. (**C**) Co-immunoprecipitation analysis of the interaction of between HK2 and VDAC1 in MDA-MB-231 cells using an anti-HK2 antibody followed by Western blotting with anti-HK2 or anti-VDAC1 antibody. V or Veh, vehicle; L or LV, lovastatin. * *P* < 0.05, ** *P* < 0.01, compared with the control.

The effect of lovastatin on the glycolytic enzymes was further examined by Western blotting. The protein levels of GLUT1, PFK1, and PKM2 were all decreased in lovastatin-treated MDA-MB-231 but not MDA-MB-453 cells compared with the control cells (data not shown). Interestingly, we found that the protein level of HK2 was not decreased but rather increased by lovastatin treatment in MDA-MB-231 cells (**Figure 4A**). We asked whether this increase in the protein level would enhance the enzymatic activity of HK. We found that although HK2 expression was increased, the total kinase activity was contrarily decreased (**Figure S4**). We thus speculate that the increased HK2 might play a non-glycolytic function.

### Lovastatin decreases colocalization of HK2 and VDAC1 in TNBC cells

Because the interaction between HK2 and VDAC1 is a key event in determining cell fate 23, we examined whether lovastatin also affected this interaction in TNBC cells. To this end, we examined the localization of HK2 and VDAC1 in lovastatin-treated and control MDA-MB-231 cells by immunofluorescence-confocal microscopy. Unexpectedly, although the protein level of HK2 was increased, colocalization of HK2 and VDAC1 was decreased after treatment with lovastatin compared with the control group (**Figure 4B**). We further used co-IP to confirm their interaction at the protein level. Consistent with the findings from immunofluorescence, co-IP assay revealed that the interaction between HK2 and VDAC1 was also decreased (**Figure 4C**). These results suggested that although lovastatin increased the protein level of HK2, its interaction with VDAC1 is contrarily decreased.

### Lovastatin promotes autophagy in TNBC cells

**Figure 5:**
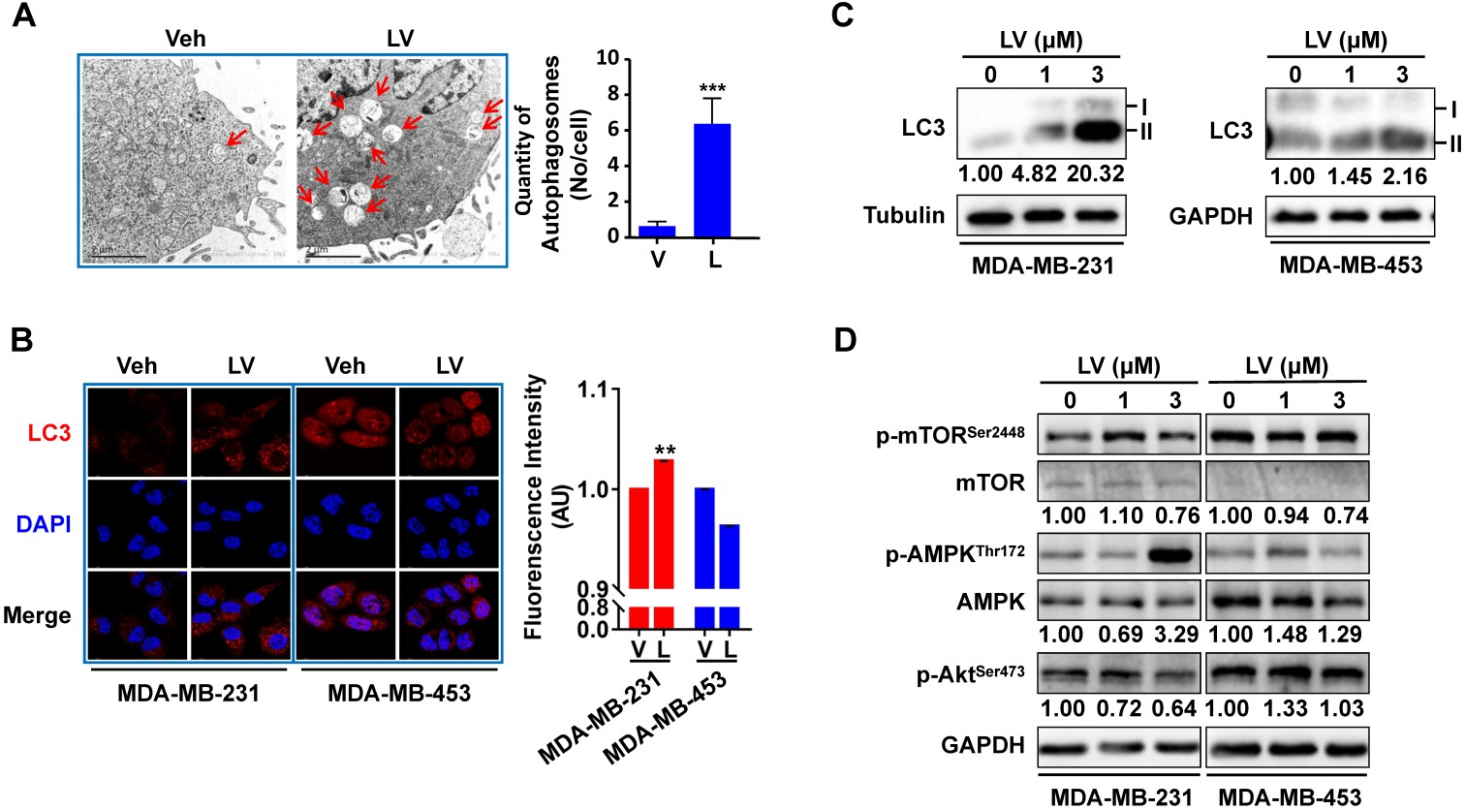
** Lovastatin promotes autophagy in TNBC cells.** (**A**) Representative transmission electron microscopic images showing the autophagosomes/autolysosomes (indicated by arrows) in MDA-MB-231 cells after treatment with vehicle or lovastatin (1 μM, 24 h). Scale bar = 2 μm. Right, quantification of the number of autophagosomes/autolysosomes per cell. (**B**) Representative confocal images of immunofluorescence staining for LC3 in MDA-MB-231 and MDA-MB-453 cells after treatment for 48 h with lovastatin (3 μM) or vehicle. Blue, DAPI staining of the nucleus. Original magnification: 630×. (**C**) Western blotting of whole cell lysates of MDA-MB-231 cells and MDA-MB-453 cells for LC3. (**D**) Phosphorylated *vs*. total mTOR and AMPK after treatment for 48 h with lovastatin at different concentrations. Numbers under the blots are quantifications of band intensities expressed as LC3-II/I ratio or phosphorylated *vs*. total protein ratio. V or Veh, vehicle; L or LV, lovastatin. * *P* < 0.05, ** *P* < 0.01, compared with the control.

To investigate whether lovastatin promotes autophagy in TNBC cells, we first performed transmission electron microscopy to reveal the presence of autophagosome/autolysosome in TNBC cells. Lovastatin indeed triggered autophagy in MDA-MB-231 cells as demonstrated by increased formation of autophagosomes and autolysosomes (**Figure 5A**). Increased LC3 puncta formation was observed by immunofluorescence-confocal microscopy in lovastatin-treated MDA-MB-231 cells but not MDA-MB-453 cells (**Figure 5B**). Consistent with the results of immunofluorescence, lovastatin increased the LC3-II/I ratio in MDA-MB-231 cells as revealed by Western blotting (**Figure 5C**). In addition, we demonstrated the decreased levels of mTOR^Ser2448^ and Akt^Ser473^ and the increased level of AMPK^Thr172^ in lovastatin-treated MDA-MB-231 but not MDA-MB-453 cells (**Figure 5D**).

### HK2 is associated with autophagy in breast cancer patients

**Figure 6:**
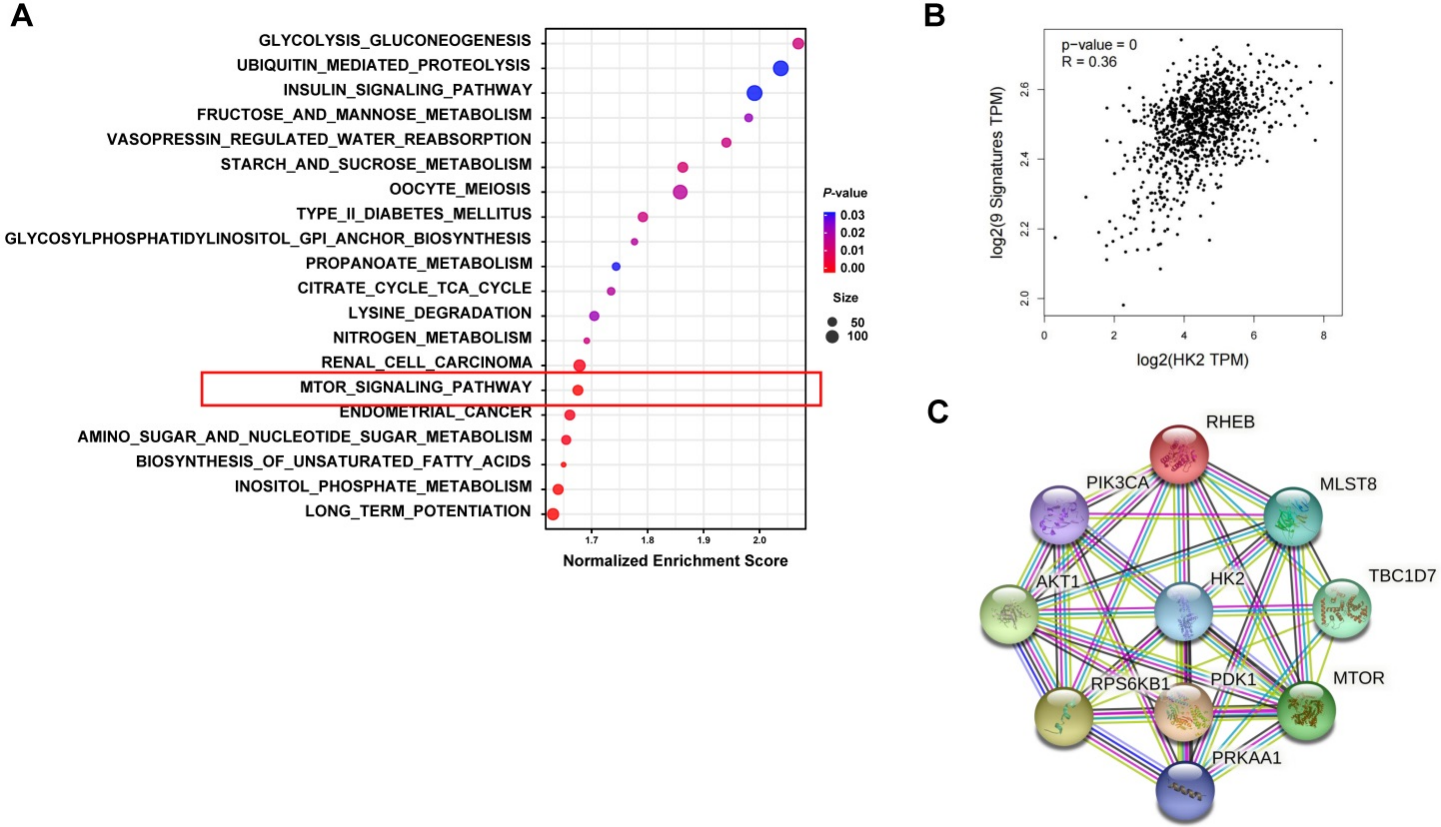
** Biological pathways related to HK2 expression in breast cancer.** (**A**) Functional enrichment of HK2^high^ expression breast cancer patients analyzed by GSEA for KEGG pathways. The mTOR signaling pathway, which is related to autophagy, is highlighted by the red box. (**B**) The correlation between HK2 and autophagy-related genes analyzed by the GEPIA2 database. (**C**) The protein-protein interaction network of HK2 and autophagy-related proteins constructed by the STRING database.

To further explore the relationship between HK2 and autophagy, we performed bioinformatics analysis comparing between the high and low HK2 expression groups in breast cancer patients. GSEA revealed that in the KEGG database, not only the glycolysis-related pathways but also the mTOR signaling pathway, which is known to be associated with autophagy, were enriched in breast cancer patients with HK2^high^ expression (**Figure 6A** and **Table S1**). Then, we analyzed the correlation between autophagy-related marker sets in breast cancer patients using the GEPIA2 and STRING databases. We found significant correlation between HK2 and the autophagy-related gene set, including mechanistic target of rapamycin kinase (mTOR), phosphatidylinositol-4,5-bisphosphate 3-kinase catalytic subunit alpha (PIK3CA), pyruvate dehydrogenase kinase 1 (PDK1), AKT serine/threonine kinase 1 (AKT1), TBC1 domain family member 7 (TBC1D7), protein kinase AMP-activated catalytic subunit alpha 1 (PRKAA1), Ras homolog MTORC1 binding (RHEB), mTOR associated protein LST8 homolog (mLST8), ribosomal protein S6 kinase B1 (RPS6KB1) in breast cancer patients through the GEPIA2 database (**Figure 6B**). Furthermore, the STRING analysis revealed functional and structural relationships between HK2 and autophagy-related proteins (**Figure 6C**). These results suggested that HK2 is indeed associated with autophagy in breast cancer patients.

### Expression levels of HK2 and VDAC1 are correlated with TNBC patient survival

**Figure 7:**
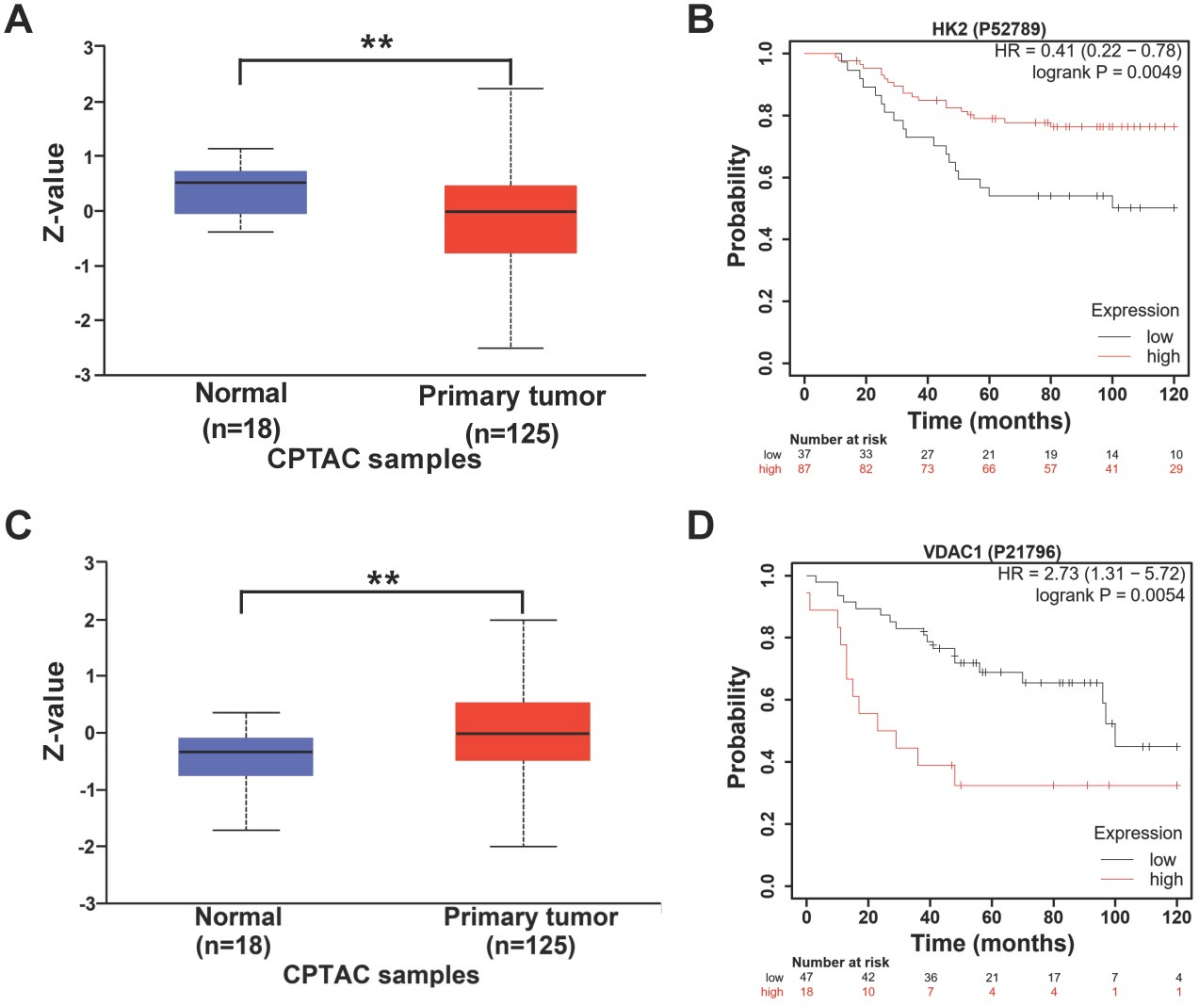
** The expression of HK2 and VDAC1 and their prognosis in breast cancer.** (**A**) UALCAN analysis for the protein level of HK2 in breast cancer patients. (**B**) Kaplan-Meier analysis revealing the relationship between the protein level of HK2 and overall survival in TNBC patients. (**C**) UALCAN analysis for the protein level of VDAC1 in breast cancer patients. (**D**) Kaplan-Meier analysis revealing the relationship between the protein level of VDAC1 and overall survival in TNBC patients.

Next, we explored the relationship between the expression of HK2 and VDAC1 and the prognosis of breast cancer patients. The protein levels of HK2 and VDAC1 and the effects on the prognosis of breast cancer patients were analyzed based on online databases. UALCAN analysis revealed that the protein level of HK2 was lower in breast cancer tissues compared with non-cancer tissues (**Figure 7A**). In addition, Kaplan-Meier analysis revealed that higher expression of HK2 protein was correlated with better overall survival in TNBC patients (**Figure 7B**). In contrast, higher VDAC1 protein level was found in breast cancer tissues compared with non-cancer tissues (**Figure 7C**) and was correlated with poorer overall survival in TNBC patients (**Figure 7D**). These results suggested that HK2 and VDAC1 are differentially expressed between breast cancer and non-cancer tissues and their expression levels are associated with TNBC patient survival.

## Discussion

It is well appreciated that lovastatin induces metabolic reprogramming in cancer cells, but current metabolic studies of lovastatin exposure are mainly concentrated on the alterations of lipid metabolism. In this study, ^1^H NMR-based metabolomics approach was adopted to explore the altered metabolites and the affected metabolic pathways after the administration of lovastatin in TNBC MDA-MB-231 cells. Out of the 46 extracellular metabolites identified, lactate, an end product of glycolysis, was obviously decreased. This indicates that metabolic reprogramming might be one of the consequences of lovastatin treatment in TNBC cells. Since the “Warburg effect” is closely associated with cancer initiation and progression, nowadays, targeting the glycolytic pathway has become one of the active research areas in cancer therapy. A promising strategy for anti-tumor therapy could be the inhibition of glycolysis-related enzymes in cancers, thus generating a state of energy deprivation that may sensitize cancer cells to other anti-cancer therapies. Statins have been reported to exert anti-tumor effects by disrupting glucose uptake and metabolism 24, 25. In our study, we found that lovastatin effectively suppressed the level of glucose uptake and lactate production and also down-regulated the expression of glycolysis-related proteins such as GLUT1, PFK1, and PKM2 in TNBC MDA-MB-231 cells.

It has been reported that the interaction between HK2 and VDAC1 predicts the roles they may have in the regulation of glycolysis and mitochondrial respiration. VDAC1, as the most abundant protein on mitochondrial outer membrane, controls the passages of metabolites, nucleotides, and ions between the cytosol and the mitochondrion 26. Our study showed that lovastatin decreased the protein level of VDAC1 and its colocalization of HK2. Many factors are important in regulating mitochondrial localization of HK2 and its interaction with VDAC1. For example, it has been demonstrated that phosphorylation of HK2 at Threonine 473 by Akt promotes mitochondrial binding of VDAC1 27, 28. In addition, Sun et al. has established that HK overexpression stimulates VDAC1 phosphorylation through a PKCɛ-dependent pathway 29. Moreover, GSK3β, of which the enzymatic activity is regulated by Akt, phosphorylates the binding site of HK2 on VDAC1, resulting in dissociation of HK2 from the mitochondrion. In this study, we found that lovastatin inhibited Akt^Ser473^ phosphorylation, which, we speculate, may be involved in enhanced phosphorylation of VDAC1 and reduced mitochondrial localization of HK2.

While the role of HK2 in autophagy has been documented in the literature, our study here represents the first observation to link glycolysis and autophagy in the case of TNBC regarding the anti-cancer effect of lovastatin. The combination of these two biological pathways might lead to new therapeutic strategies for TNBC. Tan et al. discovered that HK2 facilitates autophagy in response to glucose deprivation to protect cardiomyocytes, suggesting that HK2 functions as a molecular switch from glycolysis to autophagy 30. However, the molecular determinants responsible for the crosstalk between glycolysis and autophagy in cancer remain largely unsolved. It is possible that HK2 connects the two biological processes through the Akt/mTORC1 signaling pathway 31. Additional studies will be required to elucidate the exact role of HK2 in reprogramming glycolysis toward autophagy and to determine the mechanism underpinning this transition in TNBC cells. Our findings are helpful to understand the anti-cancer mechanism of lovastatin in TNBC cells and provide a theoretical basis for its repurposing as a therapeutic drug for TNBC, a subset of clinically challenging breast cancer.

## Supplementary Material

Supplementary figures and table.
