# Supplementary material for: Hexokinase 2 Is a Pivot for Lovastatin-induced Glycolysis-to-Autophagy Reprogramming in Triple-Negative Breast Cancer Cells
Source: J Cancer. 2022 Sep 21;13(12):3368–77. doi: 10.7150/jca.71592 (PMC9516013; doi:10.7150/jca.71592)
Supplement: Supplementary file 1 — Supplementary figures and table. [file jcav13p3368s1.pdf]

Supplementary Material

Supplementary Figures

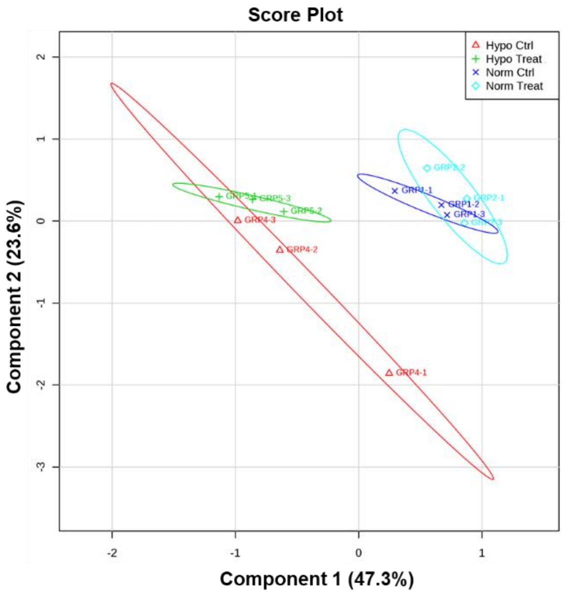

Figure S1. The PCA score plot for ^{1}H NMR data on MDA-MB-231 cells. The graph shows clear separation between normoxia and hypoxia groups, with the lovastatin-treated group and control group overlapping with each other. The samples from each group in triplicate were denoted by different colors.

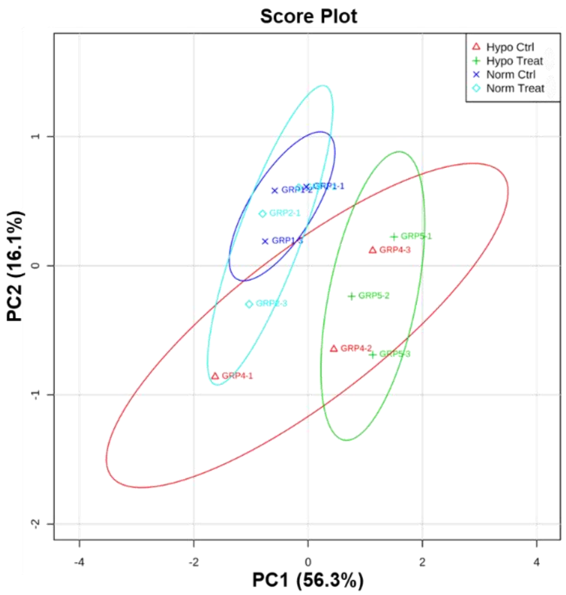

Figure S2. The PLS-DA analysis for ¹H NMR data on MDA-MB-231 cells. PLS-DA analysis of metabolomics profiles in lovastatin-treated and control groups under hypoxia and normoxia. The samples from each group in triplicate were denoted by different colors.

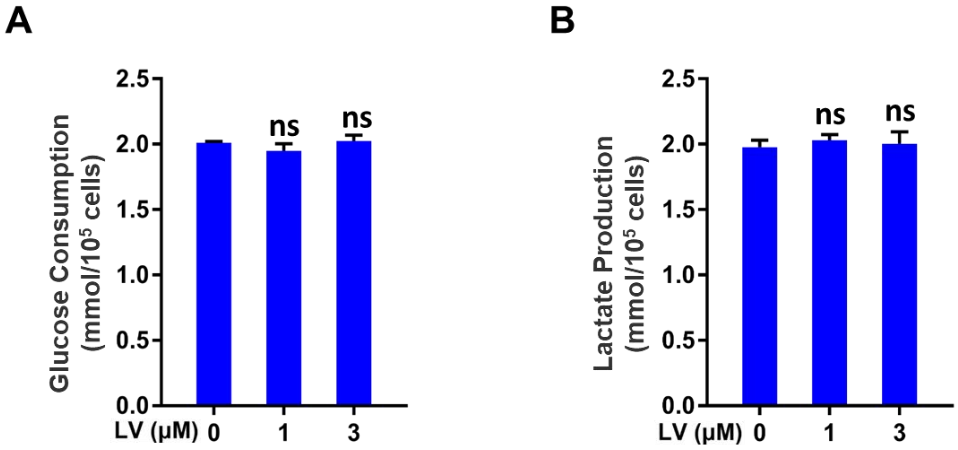

Figure S3. Effect of lovastatin on glucose metabolism in breast cancer cells. (**A, B**) The levels of glucose consumption **(A)** and lactate production **(B)** examined in culture medium collected from control and lovastatin-treated MDA-MB-453 cells after treatment for 48 h with different concentrations of lovastatin. ns, not significant compared with the control group.

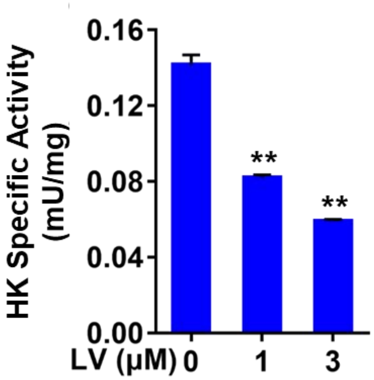

Figure S4. Effect of lovastatin on the enzymatic activity of HK in breast cancer cells. The enzymatic activity of HK examined in culture medium collected from control and lovastatin-treated cells after 48 h treatment in MDA-MB-231 cells. ** $P < 0.01$.

Supplementary Table

Table S1. Gene set enrichment analysis (GSEA) according to the HK2 expression level in breast cancer.

HK2 level	Gene set name	NES	*P*-value
High	KEGG_GLYCOLYSIS_GLUCONEOGENESIS	2.069	0.000
High	KEGG_UBIQUITIN_MEDIATED_PROTEOLYSIS	2.038	0.000
High	KEGG_INSULIN_SIGNALING_PATHWAY	1.991	0.000
High	KEGG_FRUCTOSE_AND_MANNOSE_METABOLISM	1.981	0.002
High	KEGG_VASOPRESSIN_REGULATED_WATER_REABSORPTION	1.941	0.002
High	KEGG_STARCH_AND_SUCROSE_METABOLISM	1.863	0.000
High	KEGG_OOCYTE_MEIOSIS	1.858	0.002
High	KEGG_TYPE_II_DIABETES_MELLITUS	1.792	0.014
High	KEGG_GLYCOSYLPHOSPHATIDYLINOSITOL_GPI_ANCHOR_BIOSYNTHESIS	1.777	0.023
High	KEGG_PROPANOATE_METABOLISM	1.744	0.019
High	KEGG_CITRATE_CYCLE_TCA_CYCLE	1.735	0.031
High	KEGG_LYSINE_DEGRADATION	1.705	0.019
High	KEGG_NITROGEN_METABOLISM	1.691	0.015
High	KEGG_RENAL_CELL_CARCINOMA	1.678	0.018
High	KEGG_MTOR_SIGNALING_PATHWAY	1.675	0.012
High	KEGG_ENDOMETRIAL_CANCER	1.661	0.015
High	KEGG_AMINO_SUGAR_AND_NUCLEOTIDE_SUGAR_METABOLISM	1.655	0.023
High	KEGG_BIOSYNTHESIS_OF_UNSATURATED_FATTY_ACIDS	1.650	0.032
High	KEGG_INOSITOL_PHOSPHATE_METABOLISM	1.640	0.032
High	KEGG_LONG_TERM_POTENTIATION	1.631	0.014
High	KEGG_RIBOFLAVIN_METABOLISM	1.629	0.029
High	KEGG_DORSO_VENTRAL_AXIS_FORMATION	1.623	0.027
High	KEGG_ENDOCYTOSIS	1.621	0.025
High	KEGG_PHOSPHATIDYLINOSITOL_SIGNALING_SYSTEM	1.617	0.025
High	KEGG_ALANINE_ASPARTATE_AND_GLUTAMATE_METABOLISM	1.591	0.024
High	KEGG_ERBB_SIGNALING_PATHWAY	1.560	0.043
High	KEGG_PROSTATE_CANCER	1.533	0.033
High	KEGG_CYSTEINE_AND_METHIONINE_METABOLISM	1.527	0.049
High	KEGG_COLORECTAL_CANCER	1.524	0.046
High	KEGG_NEUROTROPHIN_SIGNALING_PATHWAY	1.522	0.049
High	KEGG_LONG_TERM_DEPRESSION	1.441	0.048
Low	KEGG_BASAL_CELL_CARCINOMA	-1.602	0.042
Low	KEGG_CYTOSOLIC_DNA_SENSING_PATHWAY	-1.685	0.035
Low	KEGG_GLYCOSAMINOGLYCAN_BIOSYNTHESIS_CHONDROITIN_SULFATE	-1.727	0.026
Low	KEGG_LEISHMANIA_INFECTION	-1.756	0.042
Low	KEGG_GRAFT_VERSUS_HOST_DISEASE	-1.782	0.009

Low	KEGG_LEUKOCYTE_TRANSENDOTHELIAL_MIGRATION	-1.784	0.019
Low	KEGG_ARACHIDONIC_ACID_METABOLISM	-1.784	0.004
Low	KEGG_ALLOGRAFT_REJECTION	-1.796	0.009
Low	KEGG_VIRAL_MYOCARDITIS	-1.831	0.026
Low	KEGG_GLYCOSAMINOGLYCAN_DEGRADATION	-1.855	0.004
Low	KEGG_PRIMARY_IMMUNODEFICIENCY	-1.922	0.004
Low	KEGG_SYSTEMIC_LUPUS_ERYTHEMATOSUS	-1.945	0.006
Low	KEGG_AUTOIMMUNE_THYROID_DISEASE	-1.969	0.000
Low	KEGG_INTESTINAL_IMMUNE_NETWORK_FOR_IGA_PRODUCTION	-2.001	0.002
Low	KEGG_ASTHMA	-2.024	0.002
Low	KEGG_RIBOSOME	-2.028	0.000
Low	KEGG_CELL_ADHESION_MOLECULES_CAMS	-2.092	0.006
Low	KEGG_CYTOKINE_CYTOKINE_RECEPTOR_INTERACTION	-2.136	0.002
Low	KEGG_HEMATOPOIETIC_CELL_LINEAGE	-2.141	0.002
